# Role of Handheld In Vivo Reflectance Confocal Microscopy for the Diagnosis of Fabry Disease: A Case Report

**DOI:** 10.3390/diseases6030055

**Published:** 2018-06-27

**Authors:** Elisa Cinotti, Luca Provvidenziale, Michele Fimiani, Jean Luc Perrot, Frederic Cambazard, Pietro Rubegni

**Affiliations:** 1 Department of Medical, Surgical and Neurological Science, Dermatology Section, University of Siena, S. Maria alle Scotte Hospital, Viale Bracci 16, 53100 Siena, Italy; elisacinotti@gmail.com (E.C.); fimiani@unisi.it (M.F.); pietro.rubegni@gmail.com (P.R.); 2 Department of Dermatology, University Hospital of St-Etienne, 42055 St-Etienne, France; j.luc.perrot@chu-st-etienne.fr (J.L.P.); frederic.cambazard@chu-st-etienne.fr (F.C.)

**Keywords:** lysosomal diseases, fabry disease, confocal microscopy, diagnosis, screening

## Abstract

Fabry disease (FD) is a rare X-linked lysosomal storage disorder caused by the deficient activity of the lysosomal enzyme α-galactosidase that leads to a systemic accumulation of globotriaosylceramide. Handheld in vivo reflectance confocal microscopy (HH-RCM) is a useful modern technique in diagnosis and follow-ups of many skin diseases. This noninvasive device provides high-resolution and high-contrast real-time images to study both the skin and the ocular surface structures that can help clinicians to confirm the diagnosis of FD. HH-RCM could be helpful even for the follow-ups of these patients, enabling us to monitor the effect of enzyme replacement therapy on corneal cells and keratinocytes.

## 1. Introduction

Fabry disease (FD) is a rare X-linked lysosomal storage disorder caused by the deficient activity of the lysosomal enzyme α-galactosidase (α-Gal A) that leads to a systemic accumulation of globotriaosylceramide (Gb3). Gb3 accumulates in the vascular endothelium of skin, kidneys, nervous systems and heart, thereby leading to inflammation and fibrosis. Eventually, this process leads to multiorgan dysfunction and even death [1,2].

Early manifestations of FD include angiokeratomas (AKs), sweating abnormalities and acroparesthesis [1]. Ophthalmologic findings are also important for the clinical diagnosis of the disease. The most common ocular finding in FD is cornea verticillata (CV) [1,2,3]: corneal deposits are visible with a palm leaf pattern by slit-lamp examination. These deposits can be better observed with in vivo reflectance confocal microscopy (RCM) [3], and it has been recently reported that the handheld RCM (HH-RCM) device dedicated to dermatology is also able to identify them. HH-RCM is a useful modern technique in diagnosis and follow-ups of many skin diseases. This noninvasive device provides high-resolution and high-contrast real-time images, and it has the peculiarity of being able to study both the skin and the ocular surface structures giving details on the cellular level.

Here, we report HH-RCM corneal and skin findings of patients with FD.

## 2. Case Report

**Figure 1:**
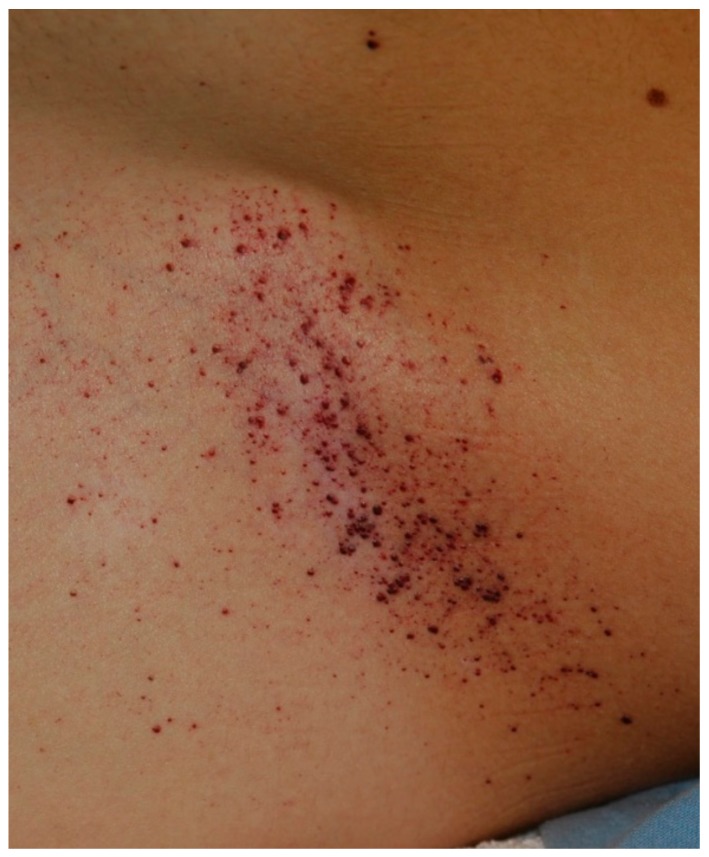
Multiple angiokeratomas on the right hip of a 33-year-old man.

A 33-year-old man presenting with multiple red-blue to black papules on his right hip and penis was referred to our clinic. Physical examination revealed multiple warty, keratotic, red-blue to black papules with a diameter of 2–5 mm on the areas mentioned above (Figure 1).

**Figure 2:**
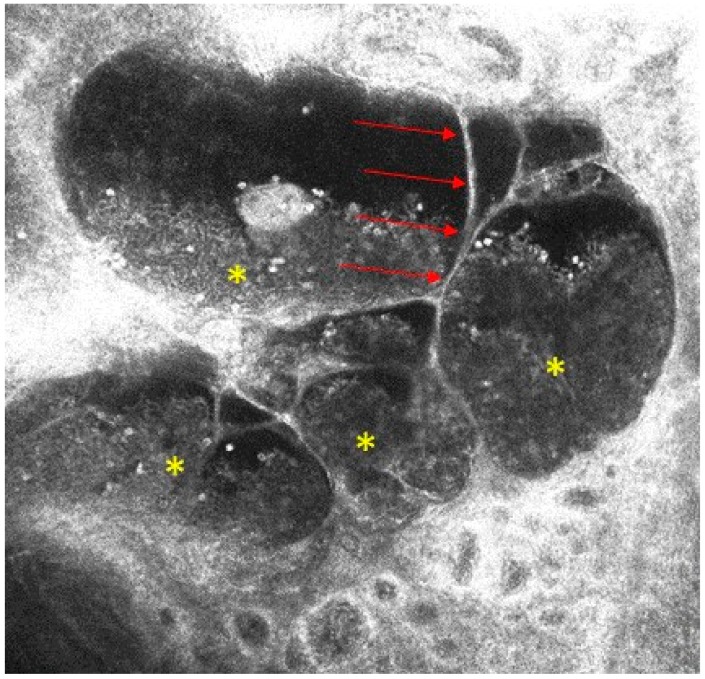
Handheld reflectance confocal microscopy examination of an angiokeratoma. Hypo-reflective oval areas separated by fine septa (→) and containing hyper- and medium-reflective cells are visible (*).

HH-RCM (VivaScope 3000, Caliber, United States, distributed in Europe by Mavig, Munich, Germany) showed an acanthotic epidermis and hypo-reflective oval areas in the dermis separated by fine septa with hyper- and medium-reflective cells floating inside. These findings were highly suggestive for dilated vascular spaces containing blood cells and supported the diagnose of multiple AKs (Figure 2).

**Figure 3:**
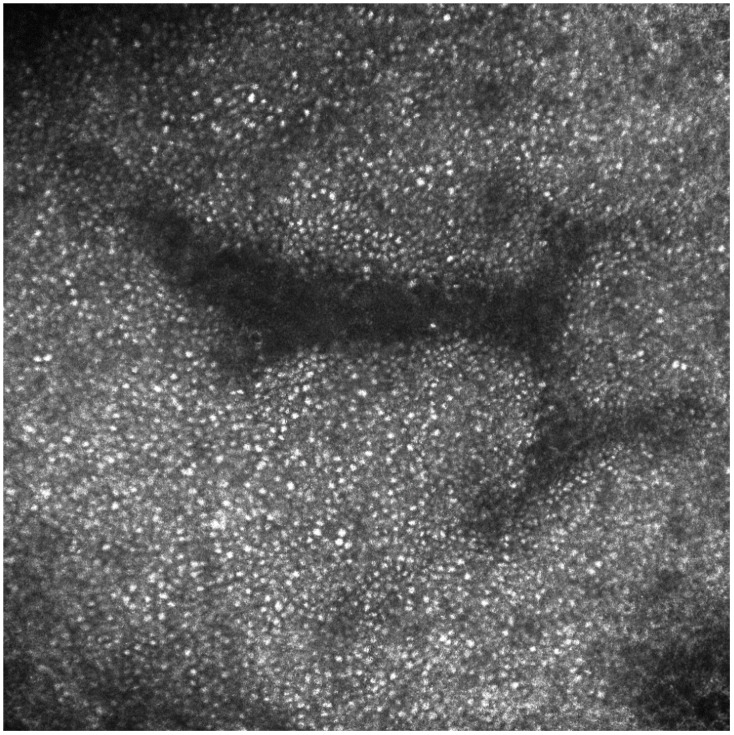
Reflectance confocal microscopy reveals the presence of intracellular hyper-reflective inclusions in most of the epithelial cells of the cornea related to deposition and accumulation of glycosphingolipids.

Slit-lamp bio-microscopy examination of the cornea showed whorl-like lines in the inferior cornea of both eyes. With a suspicion of FD, HH-RCM examination was performed after local anesthesia in order to find a corneal overload. RCM revealed the presence of intracellular hyper-reflective inclusions in most of the epithelial cells (Figure 3), which can possibly be related to deposition and accumulation of glycosphingolipids.

The demonstration of deficient α-Gal A enzyme activity in white blood cells confirmed the diagnosis of FD and the patient started the enzyme replacement therapy (Agalsidase beta). 

## 3. Discussion

FD continues to be underdiagnosed. There is still a mean delay from the onset of symptoms to the diagnosis of about one decade or more [2]. The demonstration of deficient α-Gal A enzyme activity in white blood cells of hemizygous males, the identification of a pathognomonic mutation in heterozygous females, the plasma and urine concentration of Gb3 (or Lyso-Gb3) and a biopsy examination are often necessary for FD definitive diagnosis. It is essential to recognize the early signs of FD in order to begin the replacement treatment as soon as possible.

Having a prevalence of up to 95%, CV is the most common and early ocular signs of FD [1,2,3]. Differential diagnoses include other diseases causing CV, such as lipidosis, sphingolipidosis, mucopolysaccaridosis and endothelial congenital hereditary dystrophy [3]. To the best of our knowledge, no data are available on the RCM corneal aspect of patients with other endogenous storage diseases associated with CV. However, the main cause of CV encountered in routine clinical practice is the ingestion of amphiphilic drugs, predominantly amiodarone [2]. Although amiodarone keratopathy does not differ from FD-induced CV on slit-lamp microscopy as both diseases form vortex keratopathy, it is reported in medical literature that RCM can reveal morphological differences between FD and amiodarone-induced keratopathy in most patients. Lower hyper-reflectivity, homogeneous size of the deposits and a diffuse pattern serve to differentiate sphingolipid accumulation from amiodarone keratopathy under RCM [3,4]. Our case confirmed these features since we found a diffuse hyper-reflection of all the epithelial cells of the cornea with a hyper-reflectivity that was less intense than the one identified in the case of drug-induced CV.

Multiple AKs are one of the main signs of FD. They are present in 66% of males and 36% of females with FD [1] and could be easily diagnosed by RCM dedicated to dermatology in the case of aspecific clinical presentation.

HH-RCM could be an important tool for supporting FD early diagnosis. This device can allow a real-time and non-invasive examination of both the skin and the cornea that can help clinicians to confirm the clinical diagnosis of cutaneous AKs and of corneal glycosphingolipids deposits. RCM could be helpful as a complementary tool even for the follow-ups of these patients (other parameters should be monitored including plasma Lyso-Gb3, renal and cardiac functions, skin, kidneys, and heart biopsies), enabling us to monitor the effect of enzyme replacement therapy on corneal cells and keratinocytes [3,4]. Future prospective studies are needed to compare corneal images obtained with the HH-RCM with the conventional RCM devices dedicated to ophthalmology. In addition, further studies should be performed to observe cutaneous and corneal features of patients with other endogenous storage disorders to compare them with FD.

## References

[1] Chan B., Adam D.N. (2018). A Review of Fabry Disease. Skin Ther. Lett..

[2] Hoffmann B., Mayapetek E. (2009). Fabry Disease—Often Seen, Rarely Diagnosed. Dtsch. Arztebl. Int..

[3] Wasielica-Poslednik J., Pfeiffer N., Reinke J., Pitz S. (2011). Confocal laser-scanning microscopy allows differentiation between Fabry disease and amiodarone-induced keratopathy. Graefe’s Arch. Clin. Exp. Ophthalmol..

[4] Falke K., Büttner A., Schittkowski M., Stachs O., Kraak R., Zhivov A., Rolfs A., Guthoff R. (2009). The microstructure of cornea verticillata in Fabry disease and amiodarone-induced keratopathy: A confocal laser-scanning microscopy study. Graefe’s Arch. Clin. Exp. Ophthalmol..

